# Hepatitis C Virus in mainland China with an emphasis on genotype and subtype distribution

**DOI:** 10.1186/s12985-017-0710-z

**Published:** 2017-02-23

**Authors:** Yu Zhang, Li-Min Chen, Miao He

**Affiliations:** 1 Institute of Blood Transfusion, Peking Union Medical College, Chinese Academy of Medical Sciences, Chengdu, 610052 China; 2 Sichuan Blood Safety and Blood Substitute, International Science and Technology Cooperation Base, Chengdu, 610052 China; 3 Toronto General Research Institute, University of Toronto, Toronto, ON Canada

**Keywords:** HCV genotype, Subtypes, Distribution, Co-infection, Population demographics, Antiviral treatment efficacy

## Abstract

Due to the low fidelity of the RNA-dependent RNA polymerase, Hepatitis C virus (HCV) mutates quite frequently. There are seven genetically divergent genotypes (GTs) distributed in the world, each of which contains several closely related subtypes. The peer-reviewed literatures reporting the prevalence rate of HCV GTs in Chinese hospitalized patients were identified by systematic searching of three electronic databases, and the prevalence rates were pooled through 137 qualified studies. The significant difference between HCV GT and HCV viral load and severity of hepatitis were analyzed under Chi-squared or Fisher’s exact test. Data from epidemiological studies on hospitalized patients demonstrated that HCV GTs 1–6 have been found in China, of which 1b (62.78%(95% CI: 59.54–66.02%)) and 2a (17.39% (95% CI: 15.67–19.11%)) are the two predominant subtypes. HCV GTs and subtypes exhibits significant regional divergence. In North, Northwest, Northeast, East (except Jiangxi province) and Central China (except Hunan province), HCV-1b, 2a remain the two predominant subtypes; South China shows the most abundant genetic diversity that 14 subtypes were found, and HCV-3 in the Southwest China remains higher prevalent subtype than the other regions. In addition, co-infection in Liaoning province of Northeast China is the most diverse with 10 co-infection types, and Tibet has the highest rate of co-infection. The associations between HCV GTs and patients group, severity of illness and antiviral treatment efficacy were also discussed in this review.

## Background

Hepatitis C virus (HCV) is a globally distributed hepatic virus with an estimated 130 to 150 million people (2 to 3% of the world’s population) chronically infected world widely [1, 2]. It has been reported that by 2025, HCV-related mortality will be tripled [3]. HCV infection constitutes over 70% of post-transfusion hepatitis (PTHC), and is one of the leading causes of chronic liver disease, which usually results in liver cirrhosis (LC), liver failure and hepatocellular carcinoma (HCC) within 20 to 30 years following infection [4–6]. HCV incidence in China was 0.06‰, however, in some area such as in Fujian, reached as high as 6.01% in 2010 [7]. HCV infection has become the second major type of viral liver disease only next to Hepatitis B virus (HBV) infection in China [8], and exhibits great threat to public health.

Hepatitis C virus (HCV) was classified into genus Hepacivirus of family Flaviviridae [6]. Like other positive-stranded RNA viruses, the HCV genome encodes a single polyprotein with approximately 3000-amino-acids in length with the following order: 5’-C-E1-E2-p7-NS2-NS3-NS4A-NS4B-NS5A-NS5B-3’ [9], and flanks with 5’ and 3’ untranslated regions, which consists of 341 and 27 bases, respectively [10]. HCV genome has high genetic heterogeneity with as much as 30% of sequence divergence [11]. Based on the sequence divergence, HCV was classified into seven different genotypes (GTs) and more than 90 subtypes [6, 12]. The greatest genetic diversity was observed within HCV-6, with 24 subtypes (6a-6xa) being classified [13, 14].

In China, HCV GT distribution has been associated with various geographical and demographic characteristics. Due to the increasing mobility of the population and different transmission routs, HCV GTs distribution changed gradually and co-infection with multiple subtypes as well as genetic recombinant appeared frequently in some regions. Most recently, three new subtypes have been characterized in China: HCV-1b-2a, 1b-2 k and 6d-6 k [15]. Since there is a paucity of data from large-sample study of HCV GT distribution in China, this review aims to describe the most comprehensive distribution of HCV GTs in mainland China in order to facilitate personalized treatment and to further understand the virology of HCV for vaccine and antiviral drug development in China.

## Methods

### Literature search

The literature search on HCV GT detection among clinical cases in mainland China was conducted using China National Knowledge Infrastructure (CNKI), PubMed and Wanfang Data (a Chinese professional academic database developed by Wanfang data limited-liability Company) with the key words “HCV” or “Hepatitis C virus”; “genotype” or “subtype”, “sequencing” or sequence analysis”; “transfusion transmitted disease” and “China” or “Mainland China”. No language restrictions were applied.

### Literature selection and data extraction

The inclusion criteria included: 1) The study was conducted only in mainland China (Hong Kong, Taiwan and Macao were excluded); 2) Studies involving the HCV genotype and (or) subtype distribution; 3) The study object were hospitalized patients; 4) Studies with clear sample size.

The exclusion criteria were as follows: 1) studies without exact sample size, publication year and methods; 2) Overlapping or paradoxical studies: data from literature were repeat or inconsistent between the context; 3) The study objects were blood donors or intravenous drug user; 4) HCV GTs prevalence rate in patients with HIV/HCV co-infection; 5) Comments, reviews or conference abstracts. When investigating the trends of HCV GT spatio-temporal distributions, literatures without exact study year were not included.

### Statistical analysis

Pooled results for the prevalence rate of HCV GTs and corresponding 95% CIs by metan command in Stata 12.0 were used to assess the HCV GTs distribution status among hospitalized patients in China. The % of GT in each study counted by the weighted method that the contribution of each study was determined by number of patients in the individual studies. The time trend of HCV prevalence rate, and the significant difference between HCV GT and HCV viral load and severity of hepatitis was performed using SPSS Statistics 17.0 based on Chi-squared or Fisher’s exact test of crosstabs, and *P* value <0.05 represents statistical significance.

## Results

### HCV GT distribution

#### A profile of HCV GT data

**Fig. 1:**
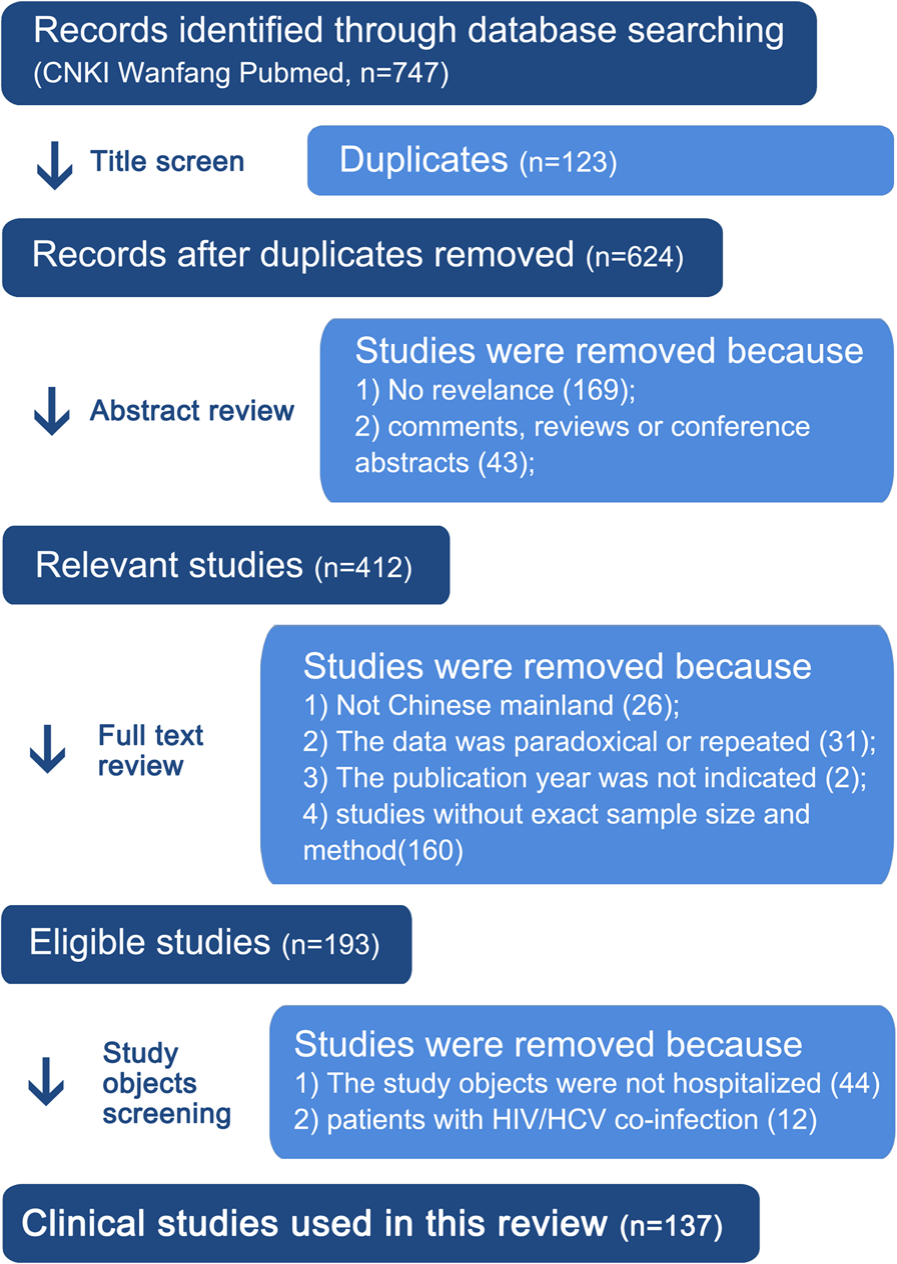
Flow diagram of summary of search strategy

Related data were collected: 193 studies [6–8, 12, 13, 16–203] considering methods used for HCV genotyping (Table 1); 137 clinical studies [7, 8, 12, 13, 16–146, 202, 203] with a total of 19712 HCV hospitalized cases considering prevalence rate of HCV subtypes [Fig. 1].

**Table 1:** Methods used for HCV genotyping in mainland China

Region	Province	Genotyping method	Amplified fragment
Central China^1^*	Hunan, Hubei, Henan	Nested-PCR and sequence analysis, PCR-RDB, GT-specific primers PCR method, gene chip assay	Core and E1, NS5B and/or Core-E1, NS5B, 5’UTR, core
East China^2^*	Shandong, Jiangsu, Anhui, Jiangxi, Fujian, Shanghai, Zhejiang	Nested-PCR and sequence analysis, RFLP, GT-specific primers PCR method, gene chip assay, Genotyping detection kit (DNA sequence assay), PCR-RDB, Micro plate nucleic acid hybridization-ELISA technique, LiPA	5’-UTR, core, NS5B and/or core-E1, 5’-UTR and/or NS5B, NS5B and C/E2, core and NS5B and 5’-UTR, E2, NS5B and E2
South China^3^*	Guangdong, Guangxi, Hainan	Nested-PCR and sequence analysis, GT-specific primers PCR method, gene chip assay, PCR – RDB, LiPA	NS5B and E1, core and NS5B, E1, NS5B, 5’-UTR and NS5B
North China^4^*	Beijing, Shanxi, Tianjin, Hebei, IM	RFLP, Nested-PCR and sequence analysis, GT-specific primers PCR method, gene chip assay, PCR fluorescent probe method	Core, C/E1, 5’-UTR
Southwest^5^*	Sichuan, Yunnan, Chongqing, Guizhou	gene chip assay, RFLP, Nested-PCR and sequence analysis, PCR-RDB, Type specific probe hybridization method	5’-UTR, 5’UTR-core and E1-E2 and NS5B, 5’NCR-C and NS5B, core/E1 and NS5B, E1/E2 and NS5B, 5’-UTR and Core, core and E1, core, C/E1, NS5B
Northwest^6^*	Gansu, Sha anxi, Xinjiang	Nested-PCR and sequence analysis, RFLP, PCR-RDB,GT-specific primers PCR method,LiPA, restriction endonuclease cleaving method	E1 and NS5B, 5’NCR, NS5B
Northeast^7^*	Liaoning, Heilongjiang, Jilin	RFLP, Nested-PCR and sequence analysis, PCR – RDB, type-specific primers PCR method, gene chip assay	NS5, Core, 5’UTR, C and E1 and NS5

PCR-RDB: Polymerase chain reaction-reverse dot blot; RFLP: Restriction fragment length polymorphism

LiPA: Line probe hybridization method

1*. See reference [7, 16–30, 131, 155, 193]

2*. See reference [6, 8, 31–67, 132–139, 147–150, 156–166, 199, 201]

3*. See reference [12, 13, 67–82, 167–178, 202]

4*. See reference [83–89, 145, 151, 152, 179–182, 203]

5*. See reference [82, 90–102, 153, 179, 188–192, 194–198]

6*. See reference [103–120, 141–144, 146, 184–187]

7*. See reference [121–130, 140, 154, 183]

Commonly used molecular biology methods for HCV genotyping include DNA sequence assay (a gold standard), type specific primers amplification method, restriction fragment length polymorphism (RFLP), gene chip assay, and probe hybridization. Multiple methods targeting different regions of the HCV genome have been used for classifying GTs. The most accurate method is to sequence an appropriate coding region that varies enough for phylogenetic analysis to distinguish genotypes and subtypes [204, 205]. Although the 5’ untranslated region (5’UTR) has most often been used by clinical laboratories for routine genotyping considering its high level of conservation, the 5’UTR is limited in its ability to discriminate GT 6 from GT 1 and subtypes within GTs 1, 2, 3, 4, and 6 [14, 206]. Nowadays, the three most commonly used regions for determining the HCV GT and subtypes are Core, E1, and NS5B [14, 207] with high accuracy and sensitivity.

#### HCV GT distribution among clinical cases in mainland China

The dominant HCV GTs in most Asian countries are HCV-1, 3 [208]. Although six GTs (GTs 1 to 6), 24 subtypes (subtypes 1a, 1b, 1c, 2a, 2b, 2i, 2f, 2 k, 3a, 3b, 3 k, 4, 5a, 6a, 6b, 6d, 6e, 6 g, 6 h, 6 k, 6n, 6u, 6v, and 6w), and a number of unassigned HCV variants have been detected in China, over 95% of these isolates belong to five major subtypes: 1b, 2a, 3a, 3b, and 6a [12, 177, 209–214].

**Fig. 2:**
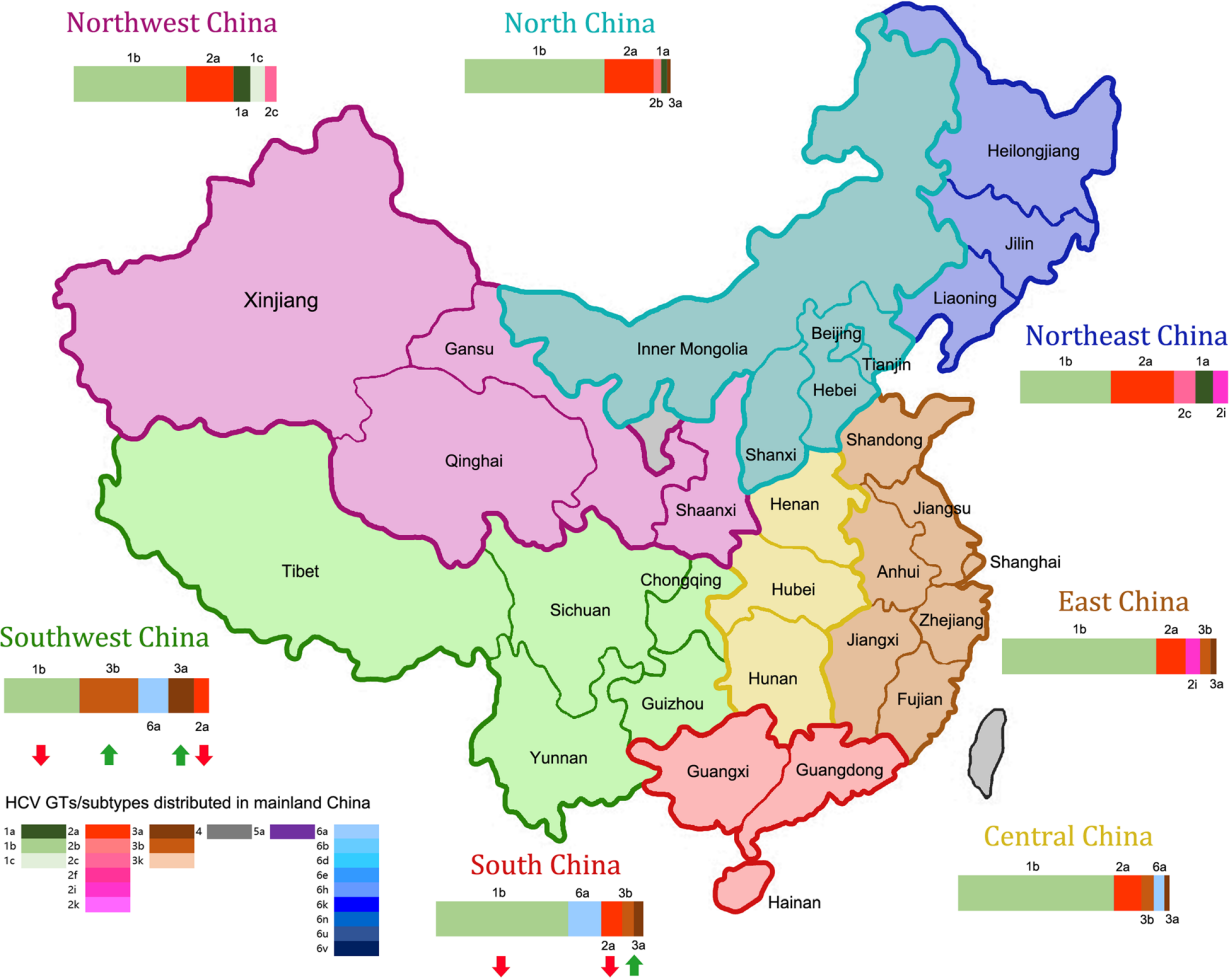
Map of HCV GT/subtypes distribution in various regions of mainland China. The seven colors in the map represent Chinese seven divisional regions; the vertical bars indicate the top five subtypes in the different region of mainland China; the horizontal bars in the lower left corner indicates the HCV genotypes and subtypes found in mainland China. The colored arrows marked in the below of genotype distribution in South and Southwest China indicates the proportion trend change of HCV genotypes with time: subtypes 1b and 2a decreased with *red arrow*, and genotype 3 increased with *green arrow*

The prevalence rate of HCV GTs in various regions of mainland China was pooled, and corresponding 95% CIs was also further tabulated (Table 2). Although HCV GTs 1–6 have been found in China, GTs 1b and 2a were the two major HCV subtypes, accounting for 62.78% (95% CI: 59.54–66.02%) and 17.39% (95% CI: 15.67–19.11%), respectively. In 70% (22/30) of provinces, HCV-1b, 2a are still the most prevalent. The major GTs in Jiangxi, Guangdong, Hunan and Guangxi were 1b, 6a, while in Yunnan, Sichuan, Guizhou and Chongqing were 1b, 3b. In Yunnan Province, 3b and 1b was the predominant HCV subtypes. HCV GT distribution also exhibits significant region divergence (Fig. 2). The major subtypes in North China, Northwest and Northeast were 1b and 2a, although 1a, 1c, 2b, 2c, 2i, 2 k, 3a, 3b, 3 k, 4, 6a were also identified. In East and Central China, HCV-1b, 2a remain the two predominant subtypes except Jiangxi and Hunan province with the popular subtypes 1b and 6a. In Southwest and South China, HCV-1b, 3b/6a was the predominant subtype. South China region showed the most abundant genetic diversity with 14 subtypes (1a, 1b, 1c, 2a, 2b, 2f, 3a, 3b, 4, 5a, 6a, 6d, 6e, 6n) being found, and HCV-3 in the Southwest remains higher prevalent subtype than other regions, which was consistent with previously published studies [212, 215].

**Table 2:** HCV GT/subtypes distribution in mainland China

Study location	Province	The dominated two GTs in each province	The other distributed GTs
East China	Shandong	1b 67.74% (95% CI: 60.94–74.53%)	6a,1a,3a,3b
		2a 27.57% (95% CI: 22.76–32.38%)	
	Jiangsu	1b 75.77% (95% CI: 72.47–79.07%)	2i, 3b, 3a, 1a, 6a, 2b, 6 h, 1c, 6b
		2a 11.01% (95% CI: 8.77–13.25%)	
	Anhui	1b 66.45% (95% CI: 47.57–85.32%)	3b, 3a, 6 k, 1a, 2b
		2a 16.52% (95% CI: 8.77–24.28%)	
	Zhejiang	1b 67.95% (95% CI: 56.70–79.20%)	3b, 3a, 6a, 1a
		2a 11.06% (95% CI: 9.06–13.05%)	
	Jiangxi	1b 70.28% (95% CI: 52.38–88.19%)	2a, 2b, 3a,3b
		6a 21.82% (95% CI: 10.90–32.73%)	
	Fujian	1b 66.17% (95% CI: 33.93–98.4%)	3b, 6a, 3a, 2b,6b
		2a 17.42% (95% CI: 11.45–23.39%)	
	Shanghai	1b 80.14% (95% CI: 69.64–90.63%)	3a,3b,1a,6n,6a
		2a 13.28% (95% CI: 8.22–18.35%)	
South China	Guangdong	1b 63.91% (95% CI: 58.48–69.34%	2a, 3b, 3a, 1a, 6e, 6n, 4, 2b, 5a,
		6a 17.32% (95% CI: 14.44–20.21%)	1c, 2f
	Guangxi	1b 56.46% (95% CI: 50.73–62.20%)	3b, 1a, 2a, 3a, 6d
		6a 12.88% (95% CI: 9.00–16.75%)	
	Hainan	1b 62.50% (95% CI: 51.32–73.68%)	3a
		2a 29.17% (95% CI: 18.67–39.67%)	
North China	Beijing	1b 70.41% (95% CI: 65.95–74.87%)	1a,2b,3a
		2a 22.15% (95% CI: 17.22–27.08%)	
	Hebei	1b 46.56% (95% CI: 40.69–52.44%)	1a,2b,3a
		2a 36.69% (95% CI: 30.05–43.34%)	
	Shanxi	1b 67.22% (95% CI: 57.44–77.00%)	1a,3a
		2a 13.51% (95% CI: 6.37–20.65%)	
	Tianjin	1b 84.21% (95% CI: 72.62%-95.8%)	
		2a 13.16% (95% CI: 2.41–23.91%)	
	Inner Mongolia	1b 63.27% (95% CI: 38.34–88.20%)	3a,1a
		2a 33.33% (95% CI: 11.97–54.70%)	
Central China	Hunan	1b 41.04% (95% CI: 33.71–48.37%)	3b,2a,3a,5a
		6a 18.50% (95% CI: 12.71–24.28%)	
	Hubei	1b 74.08% (95% CI: 66.69–81.47%)	3b, 6a, 3a, 1a,2b,6b
		2a 12.68% (95% CI: 8.91–16.46%)	
	Henan	1b 78.57% (95% CI: 62.47–94.66%)	6a,3a,3b,1a
		2a 14.26% (95% CI: 5.71–22.8%)	
Southwest	Sichuan	1b 78.84% (95% CI: 73.01–84.66%)	2a
		3b 8.47% (95% CI: 4.50–12.43%)	
	Yunnan	3b 49.50% (95% CI: 38.01–60.98%)	3a, 2a, 6n, 6a
		1b 20.52% (95% CI: 16.31–24.73%)	
	Tibet	1b 49.63% (95% CI: 41.27–58.0%)	1a
		2a 16.38% (95% CI: −5.30–38.06%)	
	Guizhou	1b 35.22% (95% CI: 30.92–39.52%)	6a, 3a, 2a, 1a, 2b, 6d
		3b 21.93% (95% CI: 18.22–25.65%)	
	Chongqing	1b 32.21% (95% CI: 25.04–39.38%)	2a, 6a, 3a, 1a, 6b, 2b,3 k
		3b 21.86% (95% CI: 7.99–35.73%)	
Northwest	Shaanxi	1b 50.74% (95% CI: 42.35–59.14%)	6a, 3a, 3b
		2a 40.39% (95% CI: 32.15–48.62%)	
	Gansu	1b 56.07% (95% CI: 49.91–62.23%)	1c, 1a, 2c, 3a, 2b, 3b
		2a 26.74% (95% CI: 16.44–37.03%)	
	Xinjiang	1b 62.71% (95% CI: 60.10–65.33%)	1a, 3a, 2b, 3b, 4, 6a
		2a 18.10% (95% CI: 11.99–24.2%)	
	Qinghai	1b 49.08% (95% CI: 30.01–68.16%)	3b, 3a
		2a 33.8% (95% CI: 26.84–40.76%)	
Northeast	Heilongjiang	1b 48.46% (95% CI: 41.29–55.62%)	2c, 2b, 1a, 3a
		2a 37.73% (95% CI: 28.82–46.65%)	
	Liaoning	1b 44.87% (95% CI: 24.30–65.44%)	1a, 2i, 3a, 1c, 2b, 3b, 3 k, 2 k
		2a 34.37% (95% CI: 11.24–57.50%)	
	Jilin	1b 56.44% (95% CI: 50.51–62.38%)	2b, 1a,3a
		2a 31.96% (95% CI: 19.42–44.50%)

HCV GT distribution reported by various Chinese regions is experiencing a remarkable change over time. An annual decrease of 1b, 2a, and an increase of GT 3a, 3b, 6n and 6a were reported in Henan, Jiangsu, Zhejiang, Chongqing, Tianjin and Yunnan [13, 16, 25, 90, 136, 152, 191, 216, 217]. We also screened qualified literatures from the hospitalized cases, and three periods (before 2005; 2005–2010; after 2010) were divided to summary respective prevalence rate of HCV GT. Comparison of prevalence rate over time was then performed to get the changing trend of HCV GT distribution. Our results showed that in Southwest and South China, HCV GT distribution experienced a significant change (*P* < 0.05) (Table 3) that the proportion of subtypes 1b, 2a were decreasing and yet GT 3 were increasing over time. In addition, HCV subtype 4, 5, 6v, 6d, 6u have also been found in some areas of China in recent years [191, 210, 211, 213, 218]. This changing trend may result from the increasing population mobility, various transmission routes, and the improved sensitivity and specificity of testing methods.

**Table 3:** Proportion change significance of HCV GT distribution with time in South and Southwest China

Region	Subtype	Year	Proportion	*P*
South China				
	1b	Before 2005	63.35% (159/251)	<0.05
		2005–2010	63.09% (400/634)	
		After 2010	55.00% (363/660)	
	2a	Before 2005	14.34% (36/251)	<0.05
		2005–2010	8.83% (56/634)	
		After 2010	9.39% (62/660)	
	3	Before 2005	6.28% (12/191)	<0.05
		2005–2010	3.55% (45/1268)	
		After 2010	8.08% (91/1126)	
Southwest	1b	Before 2005	36.99% (54/146)	<0.05
		2005–2010	18.98% (41/216)	
		After 2010	35.23% (167/474)	
	2a	Before 2005	21.92% (32/146)	<0.05
		2005–2010	6.48% (14/216)	
		After 2010	3.04% (11/362)	
	3	Before 2005	11.21% (25/223)	<0.05
		2005–2010	31.71% (137/432)	
		After 2010	18.88% (179/948)	

*P* < 0.05 indicates significant difference

#### HCV GT characteristics of co-infection in China

Presence of multiple HCV subtypes, increasing of population mobility and drug abusers, more convenient use of hemodialysis and improved detection methods increase the incidence of reported co-infection with multiple subtypes and genomic recombination. Till now, at least 5 subtypes of co-infection have been reported in China [170, 219].

In recent years, HCV GTs exhibit an increased percentage of co-infection, of which the most common in China was 1b/2a co-infection, and GTs 2a/2b and 1b/2b co-infection were occasionally reported, which may affect the host immune responses and treatment outcomes [220–222]. It was reported that among 1450 HCV RNA positive plasma (serum) samples collected randomly from general and infectious disease-specialized hospitals of the capital city from all provinces (except Taiwan), municipalities (except Chongqing), and autonomous regions (except Xinjiang), 11 was reported as HCV co-infection. These co-infection with multiple subtypes involved 1b, 2a and 6a, of which 1b/2a was the most common, accounting for 81.8% (9/11) and followed by 6a/2a and 6a/1a, which is also the first time for HCV co-infection in China that the GT 6a was found [223]. Patients who repeatedly received blood, used to share needles as intravenous drug users (IDUs) and routinely performed dialysis contributed significantly to multiple HCV genotypes/subtype co-infection in China [222, 224]. According to our statistical analysis, co-infection in Liaoning province of Northeast China is the most diverse with 10 co-infection types, and Tibet has the highest rate of co-infection, which might suggest that there had a relatively high proportion of patents who would be a repeated recipient, an IDU, or a dialysis patient. In addition, it was reported that HCV triple infections kept a relatively high percentage in Gansu, of which GTs 1a/1b/2a or 1a/1b/2c were more popular, accounting for 20.6% (14/68) infections [186].

In addition, HCV subtype 6a was most common in patients with HIV/HCV co-infection, and was mainly spread through intravenous drug use [225, 226].

### HCV GTs and different population demographics

HCV GT distribution usually varied in different populations such as in blood donors, IDUs, hemodialysis patients (HDs) and hepatitis patients. The distribution pattern changes with the change of transmission modes [227]. Generally, transfusion transmitted HCV were strongly associated with subtypes 1b and 2a [89, 180, 181]. Encouragingly, the paid blood donation is not common in China with the implementation of the Blood Donation Law in 1998. In voluntary blood donors, GTs 1b and 2a account for the highest proportion [228], and HCV GT distribution exhibits significant geographical difference: The most common GTs in Shanxi, Henan, Jiangsu, Anhui, Shanghai, Shaanxi, Hebei and Xinjiang are 1b and 2a; the prevalent GTs in Guangdong and Fujian Province are 1b and 6a; the dominant GTs in Chongqing and Yunnan are 3a and 2a/3b; GTs 1b, 1a and 1b, 3b are most common in Guangxi and Qinghai, respectively. In addition, the proportion of subtypes 1b and 2a in volunteers decreased in recent years, and yet subtypes 3 and 6 increased [179, 229]. Some reasons may explain this GT shift: 1) Increasing of drug abuser and commercial sex activity resulted in transmission from high risk population to general population; 2) Upgrade of genotyping methods accompanied by new HCV GTs and subtypes. Among IDUs, HCV-1a, 1b, 2a, 3a, 3b, 6a, 6n and 6u have been found, of which HCV-6a and 3b/3a/1b were the most popular GTs. In HD patients, the prevalence of genotype 1 is the highest, and HCV GTs among HDs are very complex with the co-infection accounting for a big proportion [224, 230, 231], which may be ascribed to long-term dialysis, repeatedly blood transfusion and iatrogenic factors. In addition, some cases of subtype 2a are found in individuals with high risk sexual behaviors, while 1b was more popular in clinical cases [194, 232, 233].

According to statistic results, discrepancy results displayed the association between HCV GTs and gender, age in infected patients. Most studies showed no statistical difference between HCV GT and gender [7, 20, 31, 136, 137, 234], while the rest displayed significant difference, of which the proportion of subtype 1b was higher in male than in female. And on the contrary, subtype 2a showed the opposite distribution [13, 32, 40, 83, 156]. The reasonable explanation for this significant difference may be that different genders exhibit different immune response against HCV infections with different GTs. For the relationship between HCV GT and age, most studies reported no statistical difference. However, there were a few studies showed that GT 1 and 2 were more common in the older group while GT 3 and 6 in the younger group [78, 137, 235]. The reason may be ascribed to be that the transmission routs are more inclined to blood transfusion in the elder group and the main GTs were HCV-1 and 2. While in the younger group, the patients were mainly infected by HCV-3 and 6 via intravenous drug abuse and high risk sexual behaviors. However, one study in 2012 showed that subtype 1 was found more often in younger patients (<40) [125], and another report showed no statistical difference between HCV-2a and age [78]. In view of the above discrepancy results, a large-scale multiple-center study involved a wider area is needed to clarify the association between HCV GT and age or gender in China.

### HCV GTs and viral loads

Many present studies were designed to determine the association between HCV GTs and viral loads in chronic hepatitis patients. It has been well known that HCV genotype/subtype together with viral load variations during treatment are very important to guide the therapeutic strategies and relapse rates [236]. However, many discrepant results were reported. Generally speaking, the higher HCV RNA loads would cause more severe liver damage with more obvious clinical manifestations [78]. The viral load in patients chronically infected with HCV is associated with GTs that the level of HCV RNA in HCV-1 patients is higher than that in GT non-1, and HCV-1 and 6 is higher than HCV-2 and 3 [52, 77, 78, 96, 152, 237], which might be due to more efficient viral replication of GTs 1 and 6 [238–240]. Some foreign studies also reported the similar results [241]. However, another study reported that among patients with high viral load in China, HCV 2a was more common than other GTs [113], while some foreign studies reported that genotype 3 or 4 were found to be significantly higher viral load as compared to other genotypes [242, 243]. In addition, higher levels of viral load were also seen in HCV co-infection [156]. Interestingly, some other studies found no significant correlation between HCV GTs and viral loads [31, 52, 78, 92, 101, 135, 153].

Considering these conflicting and complex results above, we extracted the qualified literature [17, 18, 32, 33, 44, 83, 133, 135, 152, 202, 234, 244] from the screened hospitalized patients to summary the related cases in mainland China among different GTs based on HCV RNA quantitative level (<10^6^ copies/ml indicates low viral load; ≥10^6^ copies/ml indicates high viral load), and performed the statistical analysis of between them. The overall result showed significant statistical difference (*P* < 0.05), which indicate HCV GT is associated with HCV viral load. No significant difference between subtypes 2a and 3a was found, and the significant difference was found in the other subtypes (Table 4) that HCV RNA level with subtypes 1b and 6a was higher than 2a, 3a and 3b. Various factors may be taken into account: 1) Different viral replication ability among various subtypes; 2) Regional GT distribution difference; 2) HCV infection time was not restricted exactly and identified in the same or similar stage. Since high viral load is difficult to treat than low viral load, we suggest precise mechanism study should be performed to ascertain the correlation between HCV genotypes and viral load and to further design the therapeutic strategies.

**Table 4:** Comparison of RNA levels of HCV with different subtypes

Subtypes	HCV RNA levels (lg copies/ml)	
	<10^6^	≥10^6^
1b		937
2a	208	41
3a	62	16
3b	27	66
6a		177

(1) 1b and 2a, 3a, 3b: *P* < 0.05 (2) 2a and 3a: *P* > 0.05; 2a and 3b, 6a

*P* < 0.05 (3) 3a and 3b, 6a: *P* < 0.05 (4) 3b and 6a: *P* < 0.05

### HCV GTs and severity of hepatitis

The varied HCV GTs, subtypes and strains showed different degree of liver damage [83, 245, 246]. Subtype 1b, which has more efficient viral replication ability and poor prognosis and possesses major proportion of severe liver disease (LD), is associated with HCV-related LC and HCC [83, 88, 117, 158, 247] than non-1b subtypes. The viral genome rapid replication is higher in patients with GT 1 than that in other GTs. As a result, the consequent liver histological grading and hepatitis progress speed are considerably higher than those of GT 2 infected patients, which would lead GT 1 more incline to progress into HCC consistently with previous report [87]. A study found that 5 patients with LC among 94 cases were all GT 1b [50], while 1a, 2a, 2b, 3a were more common in chronic hepatitis patients without LC and liver cancer [117, 248]. Patients infected with subtype 4 were also reported to be associated with the decompensated liver complications [249], and this strong cytotoxic effect may be associated with specific proteins encoded by subtype-specific mutant genes [250, 251]. However, other research showed that no significant differences were found between HCV GTs and outcome of HCV infections [31, 96, 128, 252]. Even so, consistent results reported presently in and out of the county were found that 1b account for a larger proportion in moderate and severe chronic hepatitis and LC than in acute, mild chronic hepatitis [253, 254], which may give us some suggestion that a proactive clinical management should be important for patients infected by HCV 1b. In addition, researches launched outside China also showed that HCV-GT 3 was associated with severe LD [255, 256].

Considering these conflicting and complex results above, we extracted the qualified literature [8, 41, 59, 65, 83, 85, 87, 96, 97, 126, 128, 140] from the screened hospitalized patients to summary the related cases in mainland China among different GTs based on severity of hepatitis (chronic hepatitis; cirrhosis; hepatocellular carcinoma), and performed the statistical analysis. The overall result showed significant difference (*P* < 0.05), which may indicate HCV GT is associated with severity of hepatitis. Significant difference was found between subtypes 1b and 2a, 3b, and the significant difference was not found in the other subtypes (Table 5). The possible reason for subtype 1b being more inclined to cause severe LD than subtypes 2a and 3b may be that subtype 1b has stronger pathogenicity and replication ability. In addition, the host factor should also be considered. Based on the above results, this may provide useful and instructional information for clinical treatment, and further mechanism study may be badly needed to clarify the association between HCV GTs and severity of hepatitis.

**Table 5:** Comparison of severity of Hepatitis with different genotypes

Subtypes	Degree of liver disease		
	chronic hepatitis	liver cirrhosis	hepatocarcinoma
1a^a^	8	1	1
1b	395	165	46
2a	228	41	13
3a	23	5	
3b	35	2	

^a^Fisher’s exact test

(1) 1a and 1b, 2a, 3a, 3b: *P* > 0.05 (2) 1b and 2a, 3b: *P* < 0.05; 1b and 3a: *P* > 0.05

(3) 2a and 3a, 3b: *P* > 0.05 (4) 3a and 3b: *P* > 0.05

### HCV GTs and antiviral treatment efficacy

In China, the current standard-of-care (SOC) for the treatment of patients chronically infected with HCV is the combination therapy of PEGylated interferon alpha and ribavirin (PEG-IFN and RBV, PR); however, HCV GT is an important viral factor to predict therapeutic response and duration of treatment [257, 258]. Various genotypes of HCV have showed different treatment effects. Patients with GT 2 and 3 can achieve relative high SVR of 70–80%, yet patients with GT 1 and 4 showed bad effect with the low SVR of 40–50% [259–262]. This may be ascribed to the different pathogenicity and replication ability of HCV with different GTs and the host cell factor [263, 264]. However, the mechanism still need to be clarified in the future, and some previous studies have reported that this may be associated with increased amount or mutation of E2 protein and the binding of NS5A protein to the protein kinase (PKR) [265, 266], a kind of antiviral protein normally induced by IFN during treatment.

With the development of new potent DAA and its combinations, the cure rate of HCV infection reaches >90% in almost all HCV GTs and stages of LD [267, 268]. Especially, Epclusa, the first oral fixed-dose combination, shows high SVR for all six major HCV genotypes, and even in patients with decompensated cirrhosis, the SVR12 can achieve 94% [269]. Actually, China has completed the clinical trials of some DAA drugs that sofosbuvir - daclatasvir or Harvoni, daclatasvir + asunaprevir, Ledipasvir/sofosbuvir and sofosbuvir + PEG/RBV are all safe and effective in Chinese patients with chronic hepatitis C [270]. Unfortunately, this DAA combination therapy is not cost-effective and for the moment, PR remains a good alternative to treat Chinese patients chronically infected with HCV with above 80% SVR which is higher than that in Europe and America [271].

## Conclusion

To sum up, HCV GTs and subtypes showed significant geographical distribution divergence in China. Along with the increasing mobility of the population, the HCV genotype distribution changes gradually. HCV GT distribution and co-infection data from our comprehensive statistical analysis in Chinese patient population provide important diagnostic and prognostic information for more effective treatment of HCV infections. In addition, more attention should be paid to intravenous drug abusing, which may have become a new risk factor for HCV transmission in China. Above all, it is urgently need to launch large-scale multi-center studies to discover the associations between HCV genotypes/subtypes and some clinical and viral factors described above. All those information are important to implement personalized and precision medicine in China for HCV infections and the relative hepatitis.

## Abbreviations

HCV: Hepatitis C virus
ETVR: End of treatment virological response
GTs: Genotypes
HCC: Hepatocellular carcinoma
HDs: Hemodialysis patients
IDUs: Intravenous drug users
LC: Liver cirrhosis
LD: Sever liver disease
LiPA: Line probe hybridization method
PCR-RDB: Polymerase chain reaction-reverse dot blot
PR: PEGylated interferon alpha and ribavirin
PTHC: Post-transfusion hepatitis
RFLP: Restriction fragment length polymorphism
SOC: Standard-of-care
